# The Kick-In System: A Novel Rapid Knock-In Strategy

**DOI:** 10.1371/journal.pone.0088549

**Published:** 2014-02-19

**Authors:** Yuko Tomonoh, Masanobu Deshimaru, Kimi Araki, Yasuhiro Miyazaki, Tomoko Arasaki, Yasuyoshi Tanaka, Haruna Kitamura, Fumiaki Mori, Koichi Wakabayashi, Sayaka Yamashita, Ryo Saito, Masayuki Itoh, Taku Uchida, Junko Yamada, Keisuke Migita, Shinya Ueno, Hiroki Kitaura, Akiyoshi Kakita, Christoph Lossin, Yukio Takano, Shinichi Hirose

**Affiliations:** 1 Department of Pediatrics, School of Medicine, Fukuoka University, Fukuoka, Japan; 2 Central Research Institute for the Molecular Pathomechanisms of Epilepsy, Fukuoka University, Fukuoka, Japan; 3 Department of Chemistry, Faculty of Science, Fukuoka University, Fukuoka, Japan; 4 Division of Developmental Genetics, Institute of Resource Development and Analysis, Kumamoto University, Kumamoto, Japan; 5 Department of Neuropathology, Hirosaki University Graduate School of Medicine, Hirosaki, Japan; 6 Department of Physiology and Pharmacology, Faculty of Pharmaceutical Sciences, Fukuoka University, Fukuoka, Japan; 7 Department of Mental Retardation and Birth Defect Research, National Institute of Neuroscience, Kodaira, Japan; 8 Department of Neurophysiology, Hirosaki University Graduate School of Medicine, Hirosaki, Japan; 9 Brain Research Institute, Niigata University, Niigata, Japan; 10 Department of Neurology, School of Medicine, University of California Davis, Sacramento, California, United States of America; University of Texas Health Science Center, United States of America

## Abstract

Knock-in mouse models have contributed tremendously to our understanding of human disorders. However, generation of knock-in animals requires a significant investment of time and effort. We addressed this problem by developing a novel knock-in system that circumvents several traditional challenges by establishing stem cells with acceptor elements enveloping a particular genomic target. Once established, these acceptor embryonic stem (ES) cells are efficient at directionally incorporating mutated target DNA using modified Cre*/lox* technology. This is advantageous, because knock-ins are not restricted to one *a priori* selected variation. Rather, it is possible to generate several mutant animal lines harboring desired alterations in the targeted area. Acceptor ES cell generation is the rate-limiting step, lasting approximately 2 months. Subsequent manipulations toward animal production require an additional 8 weeks, but this delimits the full period from conception of the genetic alteration to its animal incorporation. We call this system a “*kick-in*” to emphasize its unique characteristics of speed and convenience. To demonstrate the functionality of the kick-in methodology, we generated two mouse lines with separate mutant versions of the voltage-dependent potassium channel K_v_7.2 (*Kcnq2*): p.Tyr284Cys (Y284C) and p.Ala306Thr (A306T); both variations have been associated with benign familial neonatal epilepsy. Adult mice homozygous for Y284C, heretofore unexamined in animals, presented with spontaneous seizures, whereas A306T homozygotes died early. Heterozygous mice of both lines showed increased sensitivity to pentylenetetrazole, possibly due to a reduction in M-current in CA1 hippocampal pyramidal neurons. Our observations for the A306T animals match those obtained with traditional knock-in technology, demonstrating that the kick-in system can readily generate mice bearing various mutations, making it a suitable feeder technology toward streamlined phenotyping.

## Introduction

Knock-in mouse models are irreplaceable in research investigating heritable disease, but their production is costly and time-consuming. Furthermore, each model is committed to examining only a single variation; any additional genetic alteration, even if situated in the very vicinity of a previously examined change, requires the development of a new, separate animal line. Generating multiple knock-ins is therefore impossible, because a single animal line necessitates months of bench and colony work. Experiments comparing the effects of neighboring variations with possibly diverse outcomes are simply never started because it would require too many resources.

**Figure 1:**
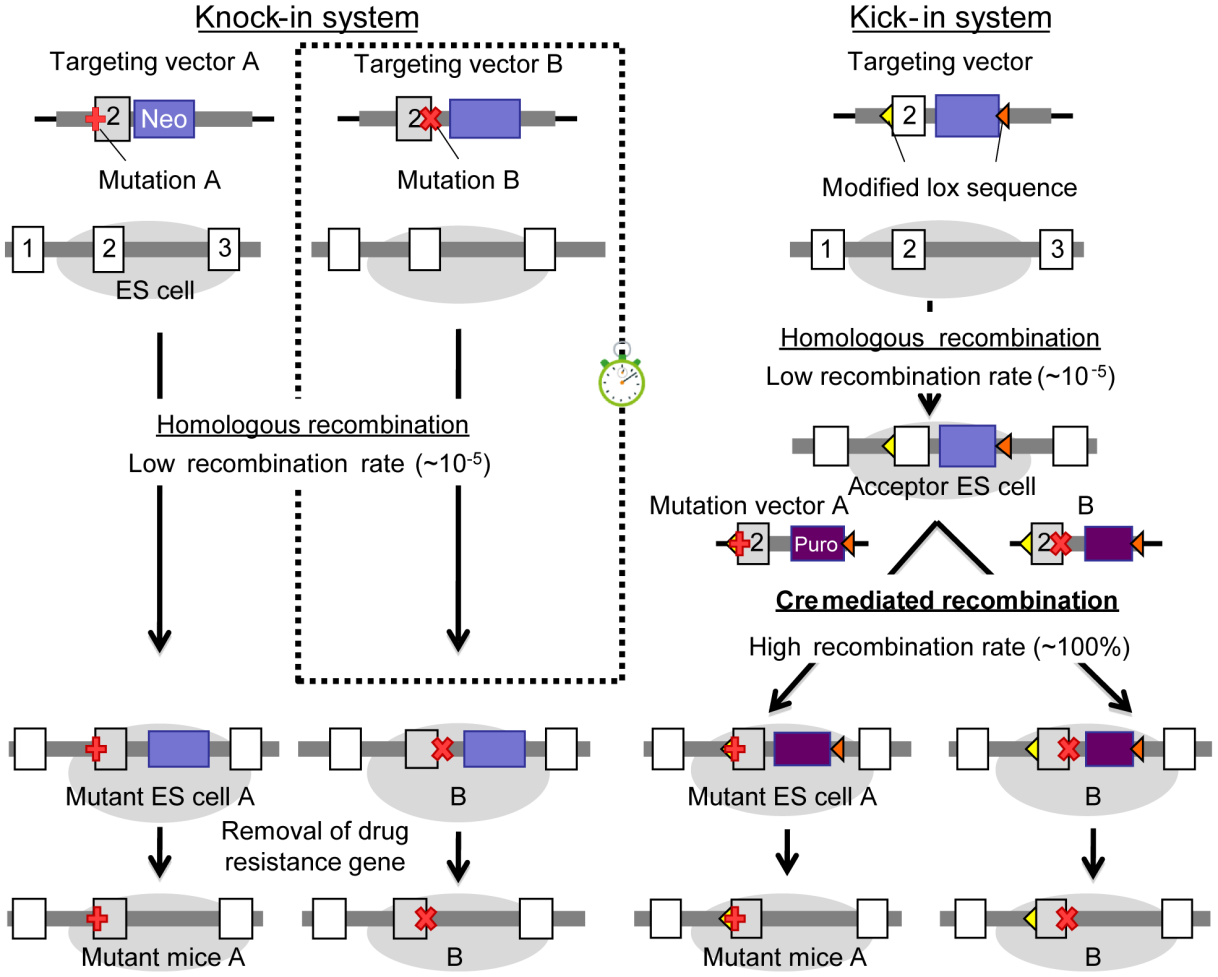
Comparison of Kick-in and Knock-in methodologies. On the left is a schematic of a traditional knock-in along with the estimated duration of each step. Juxtaposed on the right are the corresponding steps for a kick-in; savings in time and cost versus the knock-in are highlighted by a little stopwatch. Low recombination rates (∼1∶10^5^) are associated with homologous recombination. To make several constructs, homologous recombination must be performed for each construct. In the kick-in system, homologous recombination is needed only once, even if several constructs are made.

To address this problem, we revised the knock-in method to allow for the unique introduction of desired genetic alteration within a target area as illustrated in Figure 1. We call the system a “*kick-in*” to emphasize its unique characteristics in terms of speed and convenience. The kick-in method overcomes aforementioned limitations and offers knock-in technology to research requiring diverse genetic variations within a target region. To provide proof-of-principle for the kick-in strategy, we generated two mouse lines harboring separate genetic variations of the *Kcnq2* gene (protein name: K_v_7.2).

Missense mutations in *KCNQ2*, the human ortholog of *Kcnq2*, have been linked to seizure disorders including benign familial neonatal epilepsy (BFNE, OMIM #269720) [1], [2]. The prognosis of BFNE is good, because the seizures commonly decrease within months after birth [3], [4]. However, a few *KCNQ2* mutations have recently been found to cause early infantile epileptic encephalopathy, which is a malignant epilepsy phenotype [5]–[7]. Therefore, *KCNQ2* mutations induce a range of epilepsy phenotypes, and our understanding of this gene is still limited. To validate the kick-in system and to produce a new animal model for BFNE, we generated mice harboring either p.Tyr284Cys or p.Ala306Thr [8]; both mutations are located in the pore region of K_v_7.2. Knock-in mice bearing p.Ala306Thr have been previously established using conventional knock-in technology [9], which provides a suitable platform for comparison to our modifications. From our findings, we conclude that the kick-in is a suitable approach to produce several murine knock-in lines in parallel. We furthermore introduce a novel animal model for BFNE, which will help advance our understanding of infantile epilepsy.

## Materials and Methods

### Ethics statement

All animal procedures adhered to the Guidelines of the Committee for Animal Care and Use of Fukuoka University, Kumamoto University, Hirosaki University, the National Institute of Neuroscience, and Niigata University. The study was approved by the Ethical Committee for Animal Experiments of Fukuoka University, Kumamoto University, Hirosaki University, The National Institute of Neuroscience, and Niigata University.

### Vector construction

**Figure 2:**
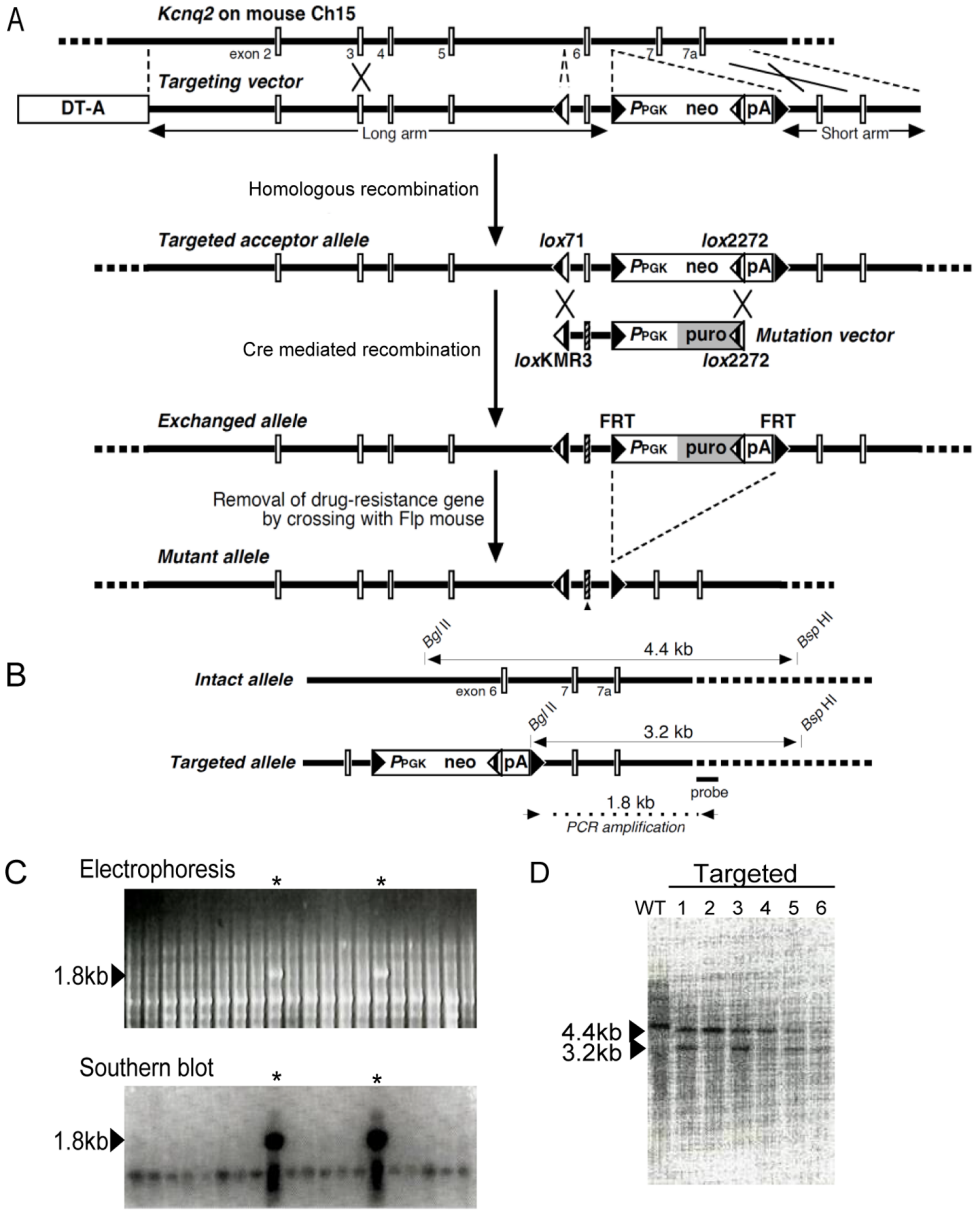
The Kcnq2 kick-in. (A) Depicted is the murine *Kcnq2* gene. Introns are shown as a solid horizontal line and the interspaced white bars symbolize the numbered exons. Aligned underneath is the targeting vector construct. Areas of homologous recombination are indicated by crosses between the two DNAs. The targeting vector, which confers neomycin resistance, introduces various *lox* elements and flippase recognition targets (FRTs) that are primed for subsequent recombination exchange with a puromycin-resistance mutation vector that contains the desired missense variations in exon 6. Triangular symbols are explained in Table 1. Additional detail is provided in the Methods & Materials. (B) Schematic explaining PCR and Southern blot analyses confirming homologous recombination in cloned embryonic stem cells. Arrows at the bottom demark the positions of primers used in the PCR analysis. Double-headed arrows span the restriction fragment used in the Southern blot analysis with the probe shown at the bottom. (C) Identification of homologous recombination clones. *Top:* PCR amplicons generated using the PCR primers that span the wildtype/recombination junction (see Panel B), such that only mutant DNA is expected to be amplified; agarose/TBE gel with the expected band at 1.8 kb highlighted with an asterisk (*); other bands result from non-specific amplification. *Bottom*: To confirm that the PCR-amplified 1.8-kb fragment originated from the recombinant *Kcnq2* allele, the DNA was analyzed by Southern blot hybridization with the probe shown in Panel B. (D) Southern blot of the genomic DNA restriction fragments from six targeted clones. Presence of a 4.4-kb signal indicates wildtype DNA, whereas 3.2-kb confirms mutant DNA (irrespective of Y284C or A306T mutation). Control setups used wildtype DNA from C57BL/6J wildtype mice.

The structures of the targeting vector and mutation vectors are shown in Figure 2; detailed sequence information has been deposited in GenBank records AB535096 and AB535097 (http://www.ncbi.nlm.nih.gov/nuccore/AB535096 and http://www.ncbi.nlm.nih.gov/nuccore/AB535097).

#### Targeting vector

Briefly, a 7.5-kb murine *Kcnq2* genomic fragment spanning from the end of intron 1 to the beginning of intron 7 was PCR-amplified from the BAC clone TRPCI23-401L17 (Advanced GenoTechs, Tsukuba, Japan); the corresponding *Kcnq2* reference sequence has the accession number NM_010611.1. The *Kcnq2* fragment was 5′-fused with a diphtheria toxin-A (DT-A) cassette PCR-subcloned from plasmid p03; additional detail is provided below. The resulting construct was modified to contain the *lox*71 sequence (Table 1) 194 bp upstream of the *Kcnq2* exon 6, and a neomycin-resistance (neo) cassette 265 bp downstream, which comprised *P*_PGK_ (promoter sequence of *mouse phosphor-glycerate kinase 1* gene), a *lox*2272 sequence, and a PGK polyadenylation sequence (pA). The *P*_PGK_-neo cassette was situated in between two yeast-derived flippase (FLP) recognition target sequences (FRTs) to allow for its removal in a flippase-enabled background. The full construct was inserted into the multiple cloning site of pBluescript II SK+ (Stratagene, La Jolla, CA). Cells harboring this targeting vector DNA after homologous recombination (“acceptor ES cells”) were primed for genetic modification within the area enveloped by the *lox*71 and the 5′-FRT (i.e., primarily exon 6). Details regarding the restriction sites and PCR primers are available upon request.

**Table 1:** Sequence information for lox sites.

Name	Sequence
*lox*P	ATAACTTCGTATA GCATACAT TATACGAAGTTAT
*lox*71	TACCGTTCGTATA GCATACAT TATACGAAGTTAT
*lox*KMR3	ATAACTTCGTATA GCATACAT TATACCTTGTTAT
*lox*71/KMR3	TACCGTTCGTATA GCATACAT TATACCTTGTTAT
*lox*2272	ATAACTTCGTATA G 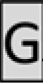 ATAC 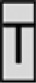 T TATACGAAGTTAT

#### Mutation vectors

To construct the mutation vectors, a 0.4-kb DNA segment containing exon 6, ranging from *lox*71 to the FRT elements of the target vector, was PCR- amplified using *lox*KMR3 and FRT primers. Fused to this fragment was a puromycin-resistance cassette (*P*_PGK_-puro) that had been PCR-amplified from plasmid p04 to contain FRT and *lox*2272. The plasmids were inserted into pBluescript II SK+, and Cre-mediated recombination produced altered alleles, in which nucleotide substitutions c.849A>G and c.915G>A (two separate mutation vectors) encoded p. Tyr284Cys and p.Ala306Thr of the mouse Kcnq2 protein, respectively. Details regarding the PCR conditions and primers are available upon request.

### Culture and gene manipulation of ES cells

Feeder-free mouse ES cells of the TT2-KTPU8 strain (C57BL/6J×CBA) F1-derived wildtype ES cell [10] were seeded at 6.0×10^6^ in collagen-coated 10-cm diameter dishes containing Glasgow minimum essential medium (GMEM; Sigma, St. Louis, MO) supplemented with 1% fetal calf serum, 14% knockout serum replacement (Gibco, Grand Island, NY), and 1 kU/mL leukemia inhibitory factor (Sigma). Following 2-day incubations at 37°C in 6.5% CO_2_ to semi-confluence (1.6×10^7^), the cells were washed twice with phosphate-buffered saline, resuspended in 1.6 mL of the same, and divided into two 0.8-mL aliquots. Transfections were performed using a Gene Pulser (BioRad, Hercules, CA) and a 4-mm gap cuvette set at 0.8 kV/3 µF in the presence of 20 µg *Sac*II-linearized targeting vector [11]. Transfected cells were expanded in G418-supplemented medium (200 µg/mL) for 9 days. Cells with randomly inserted target vector DNA did not survive during this time because non-homologous recombination failed to remove the diphtheria toxin-A cassette. Approximately 500 colonies developed, of which 144 were isolated by pipetting under a microscope, to be cultured for 3 days. Half of these cells were suspended in DMSO-supplemented culture medium and cryo-banked; the other half was used for genomic DNA extraction, followed by PCR and Southern analysis (Fig. 2C, D). A 0.2-kb DNA fragment corresponding to a partial segment of intron 7 was used as the probe for Southern blot analysis following *Bgl*II/*Bsp*HI-digestion of genomic DNA, which hybridized with 4.4-kb and 3.2-kb fragments for non-recombinant and recombinant alleles, respectively (Fig. 2B). The PCR primers Neo-3′S (sense) and KQ2TV-3′osA (antisense), which were used for detection of the recombinant allele, were designed according to the nucleotide sequences of the 3′-end of the neomycin-resistance gene and intron 7, distal to the *Kcnq2* sequence included in the targeting vector, respectively. These primers amplify a 1.8-kb DNA fragment by PCR only when genomic DNA containing homologous recombination is used as the template (Fig. 2B).

### Acceptor ES cells: insertion of mutant cassettes

Nine out of 144 clones (6.3%) were found to have recombined as desired (“acceptor ES cells”); they were used in all subsequent manipulations. After thawing, the cells were expanded in 10-cm dishes to semi-confluence, and approximately 2.5×10^6^ cells were co-transfected via electroporation (0.4 kV/125 µF) using 20 µg of uncut mutation vector and 10 µg of the Cre expression vector pCAGGS-cre. The cells were then cultured for 7 days in medium containing 2 µg/mL puromycin. From these, we isolated 12 drug-resistant colonies for each mutant. Cassette exchange was verified by genomic DNA sequencing, following PCR amplification using primers whose nucleotide sequences corresponded to the puromycin-resistance gene and short arm, respectively, and sequencing of genomic DNA.

### Development of mutant mice

Five clones of each *Kcnq2*-mutant ES cell setup were used to generate p.Tyr284Cys and p.Ala306Thr mice. To this end, we removed 2-cell stage *zona pellucida* cells from mouse embryos of the ICR line (Institute for Cancer Research), and immersed them in mutant ES cell suspension. After an overnight incubation to enhance the aggregation of the embryos and cells, the chimeric embryos were transplanted into the uteri of pseudopregnant females. From the resulting litter, checker-coated chimeras were mated with C57BL/6J mice to produce F1 heterozygous offspring with a single mutant *Kcnq2* allele. These animals were crossed with an FLP recombinase-expressing strain to remove the FRT-flanked *P*_PGK_-puro cassette. The two resulting mouse lines containing either p.Tyr284Cys or p.Ala306Thr mutations were backcrossed with C57BL/6J mice over 10 generations to establish congenic strains.

### Genotyping

Genomic DNA was extracted from the livers of 8-week-old male mice by standard proteinase K lysis and phenol extraction. A 0.4-kb region containing exon 6 was PCR-amplified (Table 2) and gel-extracted from a 2.0% agarose/TBE gel. Nucleotide sequences surrounding the mutations within the PCR products were directly determined using PCR primers and ABI-PRISM Big-dye terminator DNA sequencing on an ABI-PRISM 3100 DNA sequencer.

**Table 2:** Primer sequences.

Purpose	Sequence	Location
*Kcnq2* genomiccloning^a^	5′-GGGAAGGAGCGGCCGCAGGAAGGGGGTGGAGGGCACTGGACCTG-3′ (sense)	intron 1, *Not*I site added at 5′-end
	5′-GACGGTGCGCGGCCGCCGTGGCAGCCTGGGAAAGGCCAGAAAGAT-3′ (antisense)	intron 7, *Not*I site added at 5′-end
confirmation ofacceptor embryonicstem cells^b^	5′-CATTCCTCCCACTCATGATCTATAGATCCCC-3′ (sense)	polyadenylation region of theneomycin open reading frame inthe targeting vector
	5′-CCAGAGTCCACTGTAATTCCAAAGTCACCT-3′ (antisense)	intron 7 distal to the *Kcnq2* sequenceincluded in the targeting vector
genotypingof animals^c^	5′-TAGGGGAGCCTTGGGAATGGTTCCCC-3′ (sense)	intron 5 distal to the *lox*71 siteincluded in the targeting vector
	5′-GTATAGGAACTTCAGAGCGCTTTTGAAGC-3′ (antisense)	FRT site
amplification of*Kcnq2* transcriptin animal tissues^d^	5′-GTAGTCTACGCTCACAGCAAGGAGCTGGTG-3′ (sense)	exon 4/exon 5 junction
	5′-AGAATCTCCAGGCAGACTGGATCAGACCTG-3′ (antisense)	exon 7/exon 8 junction

a amplicon size: 7.5 kb.

b expected amplicon size: 1.8 kb; non-homologously recombined DNA is not expected to amplify.

c expected amplicon size: 0.7 kb; wildtype allele is not expected to amplify.

d expected amplicon size: 0.35 kb; wildtype and mutant transcripts were simultaneously amplified.

All primers were synthesized by GeneNet (Fukuoka, Japan).

Total RNAs were extracted from the whole brain, heart, lungs, liver, spleen, kidneys, testes, and skeletal muscle of 5-week-old male mice by using the acid guanidinium-phenol-chloroform method [12]. Synthesis of cDNAs from these RNAs was performed using PrimeScript reverse transcriptase and oligo (dT)_18_ primers (Takara Bio, Kyoto, Japan). Next, we PCR-amplified a 0.35-kb fragment surrounding the mutation using sense and antisense primers upstream and downstream of exon junctions 4/5 and 7/8 to avoid amplification of genomic DNA. Parts of the PCR products were subcloned into pBluescript II SK+ and 100 clones were selected for sequence determination. The nucleotide sequences of the PCR products and their plasmid clones were determined using PCR primers and automated Big-dye termination sequencing.

### Morphological studies

To investigate expression of the KCNQ2 protein in the brain, 4-week-old Y284C heterozygous (n = 3) and homozygous mice (n = 3) and their wildtype male littermates (n = 3) were anesthetized with sodium pentobarbital (50 mg/kg, i.p.) and transcardially perfused with 0.1 M phosphate-buffer followed by 4% paraformaldehyde in 0.1 M phosphate-buffer. The brains were quickly removed, immersed in the same fixative over night, and stored in 30% sucrose containing 0.1 M phosphate-buffer at 4°C. Vibratome sections (50-µm thick) were cut from the frontal cortex/striatum and hippocampus/thalamus, immersed in 1% H_2_O_2_ for 15 min, and blocked with 5% normal goat serum for 30 min. The sections were then incubated with polyclonal rabbit anti-KCNQ2 antibody (1∶250, ab22897; Abcam, UK) for 24 h at 4°C, followed by incubation with a biotinylated secondary antibody (1∶200; Vector Laboratories, Burlingame, CA) for 1 h and avidin-biotin peroxidase complex (1∶200; Vector Laboratories) for 1 h. The reaction was developed with diaminobenzidine (0.1 mg/mL) containing 0.0015% H_2_O_2_. For quantitative analyses, cells exhibiting somatic staining were defined immunopositive. In each mouse, we counted the number of immunopositive cells in layers II/III and V of the frontal cortex and expressed the results as cell number per unit area (1 mm^2^).

### Animal Behavior

Eight to 12-week-old heterozygous Y284C, A306T, and littermate wildtype male mice were housed at 23±2°C with a 12-h light/dark cycle; standard rodent pellets and water were provided *ad libitum*.

#### Open field

Each mouse was placed in the center of an empty 60×60 cm diameter box surrounded by 50-cm high, opaque walls. The exploratory activity and anxiety level of each animal were measured by recording the number of line crossings and spatial preference on the floor.

#### Rota rod

Defects in motor function were assessed by the animals’ ability to balance on a 10-rpm rotating 3-cm diameter rod (Neuroscience Inc., Tokyo, Japan). Latency to fall served as the operational parameter with a cut-off time of 120 s.

#### Hot plate

Abnormalities in pain perception were addressed by placing the mice on a 55±5°C hot plate encased by a 20-cm plexiglass cylinder. Measured parameters were the latency for the animal to lick its hind paws or to jump.

### Sleep and seizure analysis via video and electroencephalographic (EEG) monitoring

EEG activity and behavior were monitored in 8−12-week-old Y284C homozygous (male and female), Y284C and A306T heterozygous mutant male mice, and their wildtype male littermates over 24 h. Mice were anesthetized with sodium pentobarbital (50 mg/kg; i.p.) to implant bipolar stainless steel wire electrodes (0.5-mm diameter; Biotex, Japan) into the right forehead (+2.0 mm anterior to bregma, +1.5 mm lateral to the midline) and over the right hippocampus (−2.0/+1.5 mm). Two electromyogram (EMG) leads were placed dorsally into the neck, between the muscle and the skin. Following one week of surgery recovery, recordings of video and EEG signals continued for 24 h (Sleep Sign Version 2, Vital Recorder, Video option; KISSEI COMTEC, Japan).

### c-Fos protein expression

c-Fos expression was analyzed to investigate the neuronal hyperexcitability of the newborn pups. One to 2-day old Y284C homozygotes and heterozygotes as well as their littermate wildtype male siblings were deeply anesthetized with an intraperitoneal injection of 75 mg/kg ketamine and 15 mg/kg xylazine. This was followed by transcardial perfusion with 40 mL of heparinized (1 unit/mL) 10 µM phosphate buffer and 400 mL of 4% paraformaldehyde in phosphate buffer. The brains were extracted, postfixed in 4% paraformaldehyde phosphate buffer at 4°C for 24 h, and then infused with 15% and 30% sucrose. This was followed by coronal sectioning to 10-µm thickness using a cryostat (Leica, Wetzlar, Germany). Protein (c-Fos) was visualized using standard avidin-biotin-horseradish peroxidase immunohistochemical procedures. Tissue sections were incubated overnight with a rabbit polyclonal anti-c-Fos antibody (1∶5000; Sigma, St. Louis, MO). After washing in assay buffer (0.01 M Na^+^-phosphate), the sections were incubated in biotinylated goat anti-rabbit antibody (1∶1000; Vector Laboratories, Burlingame, CA), which was followed by incubation in an avidin-biotin-horseradish peroxidase solution. Diaminobenzidine (DAB, 0.2 mg/mL in the presence of 7 mg/mL nickel ammonium sulfate) was used as the chromogen. To allow for direct comparison of the number of immunopositive cells in different brains, all brain sections from a given experimental cohort were simultaneously processed in the same pool of each reagent using staining net dishes (Brain Research Laboratories). Cells positive for c-Fos were counted using a computerized image analysis system (Olympus Microsuite Analysis 3.2; Soft Imaging System). The threshold grey level for positive cells was set at ∼50% of the maximum grey over background. For a given brain region, 4–6 separate blinded counts were taken from separate sections/hemispheres and averaged. Two sections were used for each analysis of c-Fos immunoreactivity in the dorsal hippocampus, dentate gyrus, and somatosensory cortex. Cell counts were expressed as the number of cells per subregion (1 µm^2^).

### Drug-induced seizures

Wildtype as well as heterozygous 8 to 12-week-old male mice received 45 mg/kg pentylenetetrazole/saline (PTZ, i.p.). Seizures and behaviors were continuously recorded via EEG/video monitoring for a minimum of 30 min. Seizure scoring was based on a modified Racine scale [13].

### Brain slice preparation and electrophysiological recordings

To establish the biophysical signature of wildtype and mutant mice brains, we conducted slice electrophysiology on 4-week and 10-week-old wildtype and Y284C mutant male mice; littermates served as controls. Slice preparation followed previously established methods [14]. Briefly, mice were anaesthetized with pentobarbital sodium (25 mg/kg; i.p.) and decapitated. The brains were transferred to chilled cutting solution bubbling with 95%/5% O_2_/CO_2_ and containing (in mM): 200 sucrose, 26 NaHCO_3_, 10 glucose, 3 KCl, 2 MgSO_4_, 2 CaCl_2_, and 1.4 NaH_2_PO_4_. The brains were trimmed, mounted, and cut to 350-µm thick coronal sections using a Vibratome (VIB1500; Intracel, Royston, UK; or VT1200S; Leica, Nussloch, Germany). This was followed by a ∼1-h recovery in recording solution, which contained (in mM): 126 NaCl, 26 NaHCO_3_, 20 glucose, 2.5 KCl, 2.0 MgSO_4_, 2.0 CaCl_2_, and 1.25 NaH_2_PO_4_. The temperature was maintained at 32°C during the recording.

Whole-cell perforated patch recordings were obtained from CA1 pyramidal neurons with an Axon Instruments MultiClamp 700B amplifier (Molecular Devices, CA). Signals in the voltage-clamp was acquired at 5 kHz, low-pass Bessel filtered at 2 kHz using an Axon Digidata 1332A digitizer (Molecular Devices), and analyzed offline using pClamp 10 (Molecular Devices). Borosilicate glass capillaries (World Precision Instruments, Inc.) were pulled to 2.5–3.5 MΩ using a Flaming-Brown micropipette puller (PP-83; Narishige, Tokyo, Japan; or P-97; Sutter Instruments, Novato, CA). Input and series resistance values of 80–120 MΩ and <15 MΩ, respectively, were used as selection criteria for acceptable recordings; pipette capacitance was canceled using the amplifier circuitry. Amphotericin B (0.45–0.5 mg/mL) was dissolved in pipette solution containing (in mM) 150 K-methanesulfonate, 10 Hepes, 5 KCl, and 3 MgCl_2_ at pH7.28 (KOH). ACSF contained bicuculline (10 µM; Sigma) and CNQX (10 µM; Sigma) to block GABA_A_ receptor-mediated and non-NMDA receptor-mediated responses, respectively. Under these conditions, using animals at 4 weeks of age, the sodium and calcium currents are negligible, making their blockade unnecessary. Recordings of M-type K^+^ currents (*I*_M_) were acquired in voltage-clamp mode, using the peak amplitude of tail currents produced by voltage step from −20 mV to −40, −50, −60, and −70 mV, as previously described [14].

### Data analysis

All data are presented as means ± SEM. For parametric data, statistical analyses were performed by one-way ANOVA followed by a *post hoc* Tukey-Kramer test. Fisher’s exact test was used where appropriate. Calculations were performed using Statcel software (OMS Publishing, Japan). *P* values <0.05 were considered statistically significant.

## Results

### The kick-in strategy

Introducing a particular mutation into the genome of an animal is a time-consuming and elaborate task. The goal of this work was to develop a strategy that simplifies this undertaking. To this end, we adopted a 2-step protocol where ES cells are first transfected with a targeting vector that introduces site/orientation-directing recombination sites flanking an area of interest. These acceptor ES cells are transfected a second time to substitute the recombination-site flanked target region with mutated DNA. The production of the acceptor ES cells is the first and rate-limiting step, which lasts ∼2 months, because it employs traditional homologous recombination technology. The second step, which involves replacing the target region with a mutant construct, is guided by a highly efficient process requiring 8 weeks. This translates into marked time savings prior to phenotyping efforts, as rapid production of multiple knock-in lines becomes possible (Fig. 1).

#### Step 1: Acceptor ES cell production

The targeting vector is a key element of our approach. It provides for site/orientation-directed recombination through reconfiguration of Cre/*lox*P elements. The vector is not designed to genetically ablate a particular genomic region, but to insert an exchange acceptor site for later manipulation. To do so, we modified the *lox*P sequence by substituting 5′- or 3′-end bases that prevented self-recombination (*lo*x71 and *lox*KMR3, Table 1, boxed sequence). In combination with a third modified *lox* site, namely, *lox*2272 [11], this approach produced site-directed as well as orientation-directed recombination. The *lox*KMR3 element is a new right end-modified mutant found to efficiently recombine with *lox*71 [15]. The sequence site coming out of the recombination of these two *lox* elements contains modified bases derived from both *lox* elements. The resulting end-modified *lox*-like sequence is no longer a target of the inverse action of Cre, which ensures that the integrated mutation cassette is stably retained. Further control over recombination is provided by *lox*2272, which specifically recombines only with itself. Directional insertion of a mutation cassette (Step 2) is thus controlled by the appropriate combination of two different *lox* recombination sites. In the given scenario of 5′-*lox*71 and 3′-*lox*2272 (Fig. 2A), recombination only occurred with a fragment that was flanked by *lox*KMR3 at the 5′-end and *lox*2272 at the 3′-end [11], [16].

To simplify the selection of recombinant clones, we utilized a neomycin-resistance gene in the target vector. Expected homologous recombination on the chromosome excluded the diphtheria toxin cassette (DT-A), whereas random insertion of the target vector DNA associated with the DT-A. Therefore, only recombinant clones survived, and clones containing random insertions were killed by diphtheria toxin (see kick-in example in Fig. 2A).

#### Step 2: Acceptor ES cell mutation

Following Step 1, mutant DNA fragments were inserted into the ES cells. This was achieved by transfecting the acceptor ES cells with the respective mutation vector(s) comprising the *lox*KMR3 (5′) and *lox*2272 (3′) sites up- and downstream of the target region. Successful insertion was monitored via drug resistance to puromycin (Fig. 2A). Successful recombination was confirmed via PCR and Southern blotting (Fig. 2B–D). Undifferentiated cells were implanted into pseudopregnant females, and the offspring were backcrossed with FLP-expressing animals, to remove the FRT-enveloped puromycin cassette [17], [18]. The resulting litter carried wildtype DNA with the exception of the desired genetic alteration, as well as two novel recombinase elements, a *lox*71 element and an FRT site, which occurred in intronic areas upstream and downstream of the target.

### Proof-of-principle: *kcnq2* mutation kick-in

**Figure 3:**
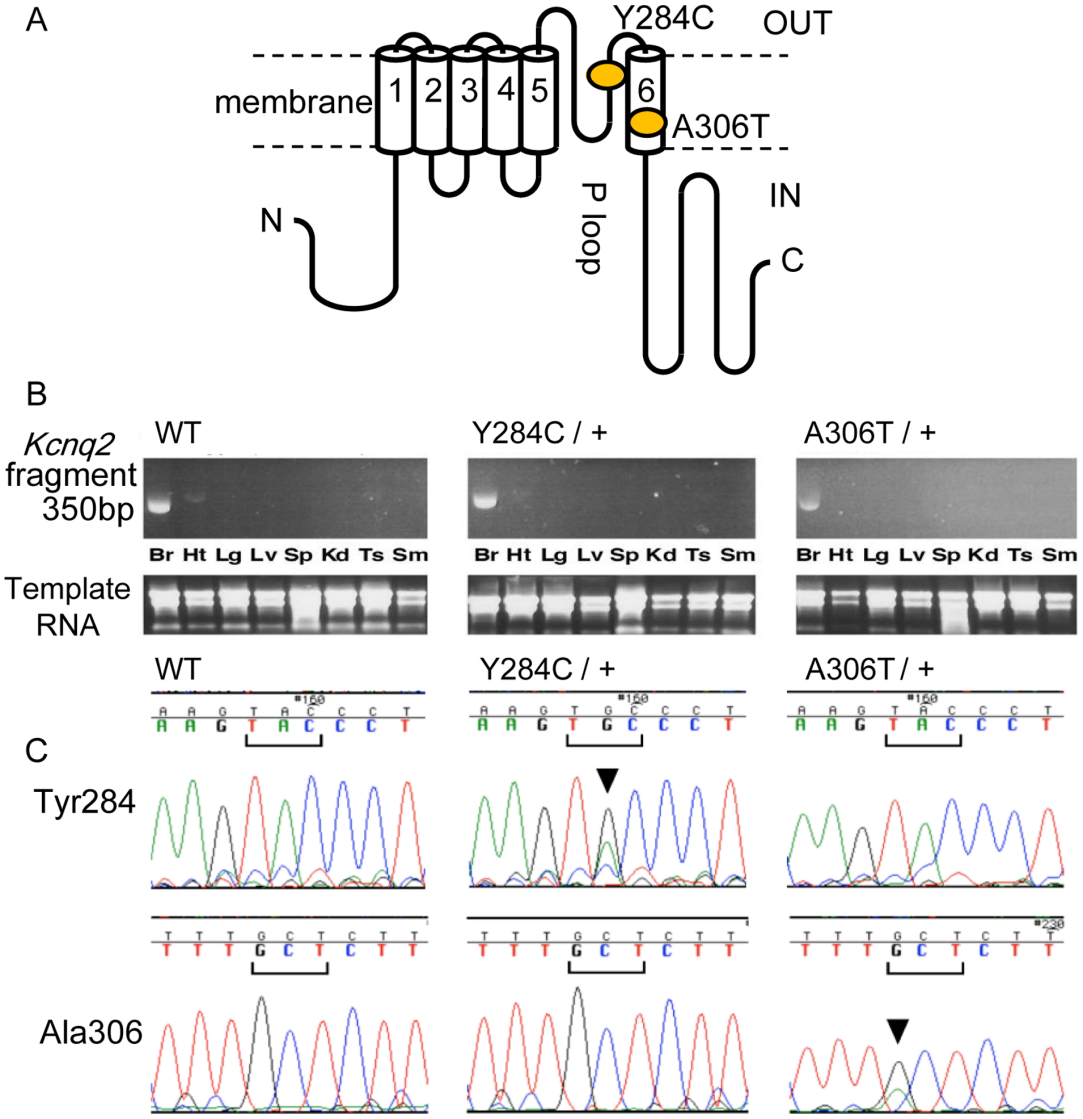
Genotyping of Kcnq2 mutant mice. (A) Based on structural analogies to related channels, p.Tyr284 and p.Ala306 map to the outer mouth of the pore and the inner lining of the channel, respectively. (B) Expression specificity of the mutated *Kcnq2* alleles. Exon 6, including 350-bp segments of *Kcnq2* transcripts from various tissues, was amplified by RT-PCR and electrophoresed on 2.0% agarose/TBE gels (top). Reference RNA samples are shown at the bottom. Br, brain; Ht, heart; Lg, lung; Lv, liver; Sp, spleen; Kd, kidney; Ts, testes; Sm, skeletal muscle. (C) Nucleotide sequence of wildtype and knock-in DNA. Direct sequencing of a PCR fragment amplified from genomic DNA using primers complementary to the *lox*71/KMR3 site and the FRT site, which are 5′ and 3′ of exon 6, respectively. Depicted are partial chromatograms for nine bases that cover the codon of interest, as well as one upstream and one downstream codon at position p.Tyr284 (top) and p.Ala306 (bottom). From left to right are sequencing results for wildtype, and heterozygotes of Y284C and A306T. Arrowhead, mutated base.

To demonstrate the workflow and efficiency of the kick-in approach, we generated two mouse lines with separate *Kcnq2* mutations, Y284C and A306T (Fig. 3A). We chose these two particular variants because A306T already exists as an animal model, making it an excellent choice for a direct comparison to current knock-in technology. The second variant, Y284C, has not been introduced into animals so far; it is a novel murine model for BFNE.

The *Kcnq2* kick-ins began with the target vector construct shown in Figure 2A containing *Kcnq2* DNA spanning from intron 1 to intron 8 (Fig. 2A). Selection with neomycin produced cell colonies that were screened for homologous recombination by PCR. The forward primer was complementary to the target vector sequence, and the reverse primer was situated in the adjacent wildtype region (Fig. 2B, Table 2). This produced amplicons with the expected 1.8-kb length for 9 of the 144 isolated colonies (1.8% of the originally isolated 500 colonies, Fig. 2C). Southern blotting with a radiolabeled probe confirmed that the 1.8-kb amplicon was indeed of the desired nature (Fig. 2C, *bottom*). The resulting positive clones were additionally subjected to restriction analysis using a *Bgl*II/*Bsp*HI double-digest of genomic DNA, followed by Southern blotting as described above. Wildtype DNA produced a signal at 4.4-kb (Fig. 2D, wildtype lane), whereas recombinant samples yielded a 3.2-kb fragment (Fig. 2D, lanes 1–6). Cell clones showing all the appropriate results were deemed “acceptor ES cells”.

We next inserted Y284C and A306T-mutant DNA. Acceptor ES cells were electroporated with the respective mutation vectors and grown in puromycin-supplemented medium to select for mutation-recombinant clones (Fig. 2A). Following PCR recombination analyses as described above, five morphologically undifferentiated clones were brought to term using standard procedures. The offspring were mated with FLP-expressing animals, thereby yielding two lines with Kcnq2 Y284C and A306T. Inheritance of the mutant *Kcnq2* allele was verified in each generation via genomic DNA extracted from the tails of 4-week-old mice. Mutant alleles were detected by PCR amplification of a 0.7-kb segment between the intron 5 sequence and FRT elements. As expected, the wildtype setups did not produce any PCR amplicons.

To determine where the mutant allele was expressed, we used RT-PCR with RNA from brain, heart, lungs, liver, spleen, kidneys, testes, and skeletal muscle of 8-week-old mice. The PCR setups following reverse transcription used primers that were designed to cover exon junctions 4/5 and 7/8, producing an amplicon of 350 bp. In wildtype mice and both kick-in mice, the patterns were consistent with expression that is limited to the brain (Fig. 3B). Some faint banding was detected in the heart of the wildtype as well as Y284C, but not A306T. The wildtype also produced a very faint signal in the testes, which was not discernible in either one of the two knockouts.

### Morphological studies

**Figure 4:**
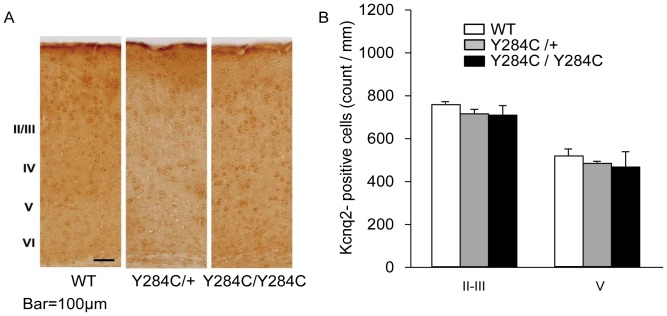
Morphological studies. (A) Light micrographs of K_v_7.2 immunoreactivity in the sensorimotor cortex. Note the immunostained neuronal cell bodies in layers II to VI. (B) Cell count of K_v_7.2-immunopositive neurons in layers II/III and V in the frontal cortices of wildtype, heterozygous, and homozygous Y284C mice. No differences were noted.

There were no brain anomalies detected in any of the animals. In the sensorimotor cortex, immunohistochemical analysis revealed no differences in expression of the Kcnq2 protein between Y284C/Y284C, Y284C/+, and wildtype mice (Fig. 4A). Furthermore, there was no significant difference in the number of Kcnq2-immunopositive neurons between Y284C/Y284C, Y284C/+, and wildtype mice (Fig. 4B).

### Behavioral and eEG analyses of *kcnq2* mutants

**Figure 5:**
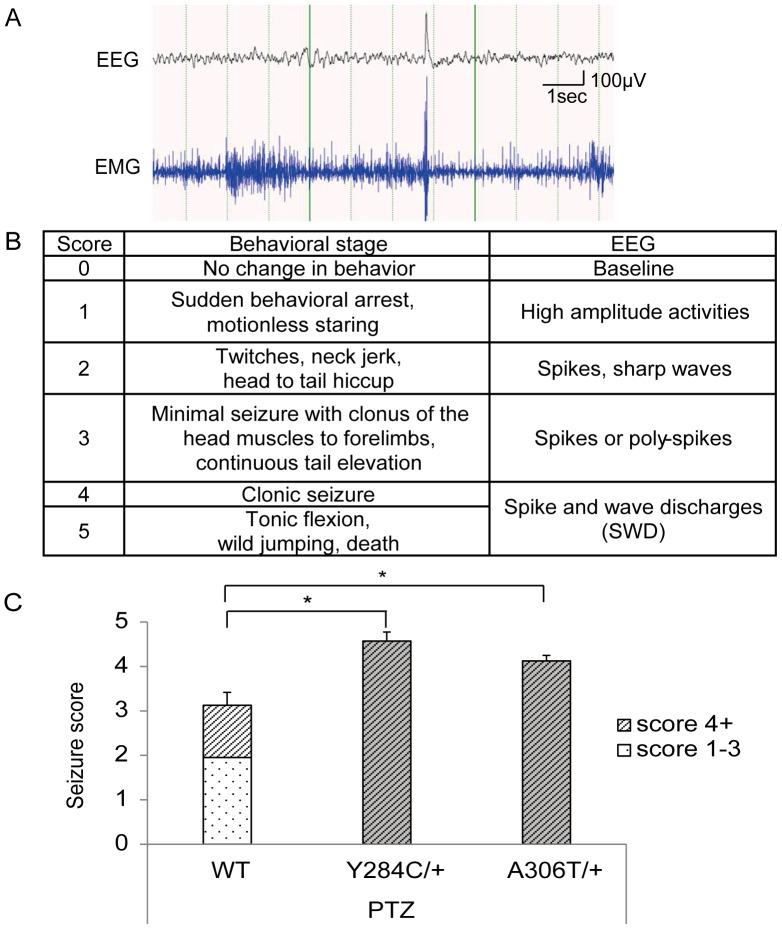
Behavioral analysis of Kcnq2 kick-ins. (A) Electroencephalography (EEG) with video monitoring revealed spontaneous myoclonic seizures with spike discharges in all Y284C homozygotes (n = 4). On average, the animals had 12 myoclonic seizures per hour in the daytime. (B) Modified Racine’s scale. Behavioral criteria used to score the seizures in this study. Adopted and modified from Lüttjohann et al., 2009. All seizures scoring 4 or above were generalized. (C) Pentylenetetrazole/saline (PTZ) induced seizures in wildtype (white bar), Y284C, and A306T heterozygous mice (grey bars). The bar height represents the average seizure score; the hashed part of the bar represents the fraction of the animals that reached seizure score 4 and above. Asterisks denote *P*<0.05 (Tukey-Kramer test).

Analyses using an open field setup, rotarod, and hot plate showed no significant differences between the wildtype and heterozygotes with respect to general behavior and sensorimotor function (n = 8–10; Tukey-Kramer test). Heterozygous animals of both lines displayed normal behavior and the EEG was unremarkable. Sleep ratios for non-rapid eye movement sleep, rapid eye movement sleep, and awake state were indistinguishable between wildtype and heterozygous animals (n = 8–10; Tukey-Kramer test). On the other hand, some of the Y284C homozygotes showed spontaneous seizures 6 weeks after birth. This included generalized forelimb and hindlimb clonic seizure followed by arrest with salivation (one female) as well as weak myoclonic seizures with spike discharges in the EEG. All Y284C homozygous mice (n = 4) showed these seizures with an average of 12 myoclonic attacks per hour in the daytime (Fig. 5A). Animals homozygous for A306T displayed no seizures, but died shortly after birth, making EEG analysis impossible.

### Expression of c-Fos in *kcnq2* mutants

Since neither of the heterozygous kick-in mice presented with spontaneous seizures but BFNE is characterized by neonatal seizures, we asked whether very early seizures had been missed prior to weaning. Evidence of such seizures can be produced postictally by examining c-Fos expression. We compared brain c-Fos expression in wildtype and Y284C mutant animals, because this protein acts as an indirect marker of neuronal seizure activity [19], [20]. In the somatosensory cortex, no differences were noted with respect to c-Fos-positive cells. Comparatively, in the dorsal hippocampus of homozygous mutants, c-Fos expression was markedly elevated versus wildtype and heterozygous Y284C animals (Fig. S1; *P*<0.05; n = 3–8; Tukey-Kramer test), suggesting abnormal excitation levels. In the dentate gyrus of heterozygous Y284 animals, a large percentage of cells were c-Fos-positive, albeit without reaching statistical significance. None of the other brain regions examined showed any discernible difference compared to wildtype animals. Taken together, these data indicate that neuronal hyperactivity occurred not only in the homozygous animals, but also in the heterozygous animals, which did not present with spontaneous seizures.

### PTZ-induced seizures in heterozygous mice

We challenged the mutant animals with the chemical convulsant PTZ (45 mg/kg; i.p.) and scored them on a modified Racine scale (Fig. 5B). Heterozygotes injected with PTZ showed a significant increase in seizure severity compared to wildtype animals. Seizure scores averaged 4.5±0.2 and 4.1±0.1 for the Y284C and A306T mice, respectively (*P<*0.01 vs. WT, n = 6–8; Tukey-Kramer test), and 3.1±0.3 in the wildtype animals (Fig. 5C). We also examined the seizure type, based on an operational threshold of seizure level 4+ (shown as a fractional pattern in Figure 5C). Heterozygotes reached score 4 seizures in all cases, compared to 4/10 animals (38%) in the wildtype (Fig. 5C; *P<*0.01 for Y284C and A306T; Fisher’s exact test). Inspection of the seizure latency revealed no differences between wildtype and both groups of heterozygous mice.

### M-current in hippocampal pyramidal neurons

**Figure 6:**
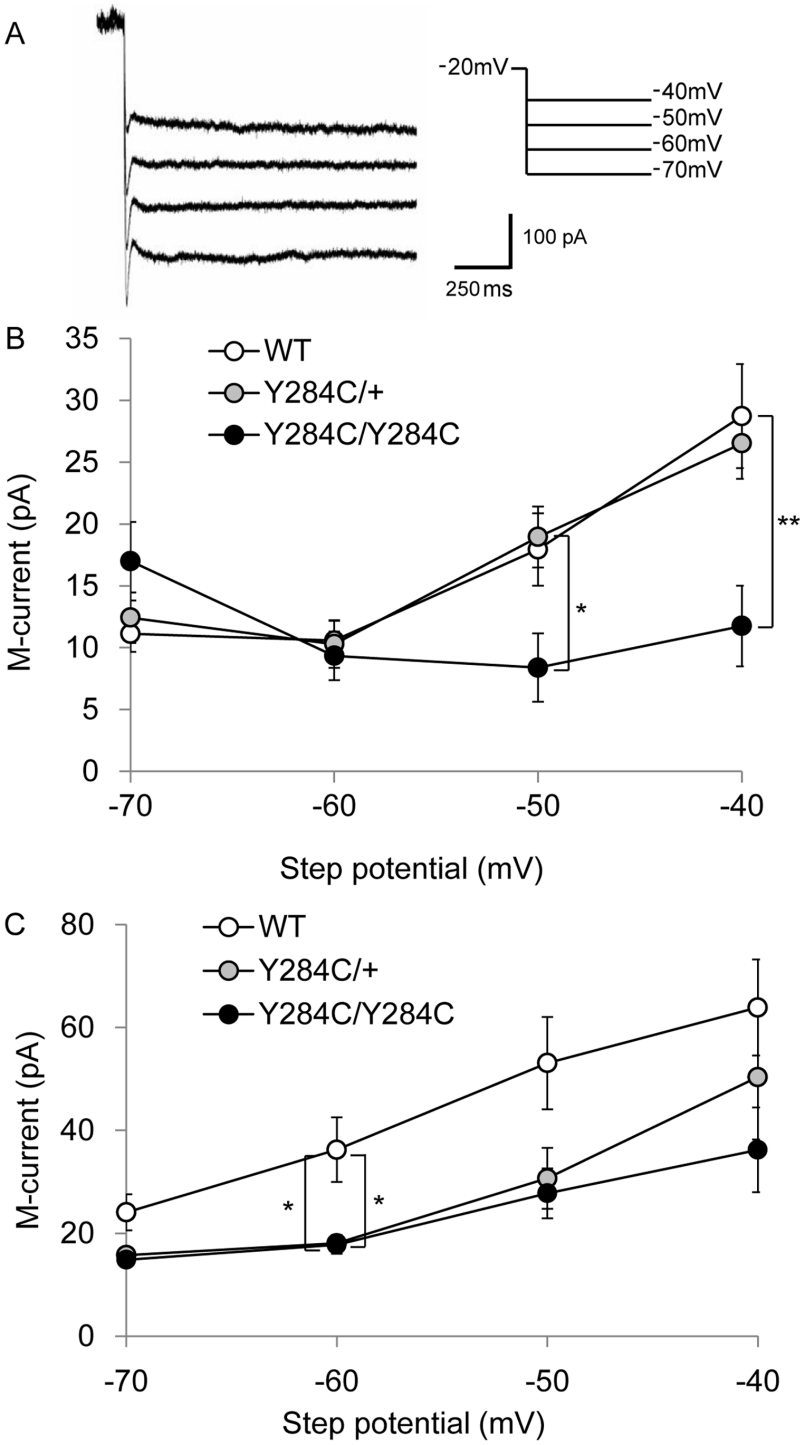
Kcnq2 mutant brain slice electrophysiology. (A) Sample traces recorded from 10-week old Y284C heterozygous CA1 neurons in response to voltage steps to the indicated potential. (B, C) Voltage-dependence of M-current activation in wildtype (○), heterozygous (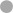), and homozygous (•) Y284C mice at 4 weeks (B) and 10 weeks (C). Whole-cell perforated patched CA1 neurons were stepped to the indicated potential and back to −20 mV to determine tail current amplitudes. Significant *I*_M_ reductions were seen in Y284C homozygotes at 4 and 10 weeks, and in heterozygotes at 10 weeks at −60 mV (*P*<0.05, Tukey-Kramer test).

Since central hyperexcitability was evident in the mutant mice, we examined the electrophysiological properties in brain neurons of these animals. More specifically, because K_v_7.2 plays a role mediating M-current (*I_M_)*, we voltage-clamped CA1 neurons in the perforated patch configuration to examine the biophysical signature of the hyperpolarizing K^+^ current. We measured the tail current amplitude by stepping from the holding potential (−20 mV) to various test potentials (from −70 to 40 mV, Fig. 6A). At more depolarized potentials, we noticed a significant *I_M_* reduction in homozygous Y284C mice aged 4 and 10 weeks, and in 10-week- old heterozygous Y284C mice at −60 mV (Fig. 6B, C); 4-week-old, −40 mV: wildtype = 28.7±4.2 pA (n = 30); Y284C/+ = 26.5±2.7 pA (n = 39); Y284C/Y284C = 11.8±3.3 pA (n = 20); F = 5.31, *P* = 0.0067 by one-way ANOVA, post-hoc, wildtype *vs.* Y284C/Y284C, *P*<0.01 by Tukey-Kramer test. 10-week-old, −60 mV: wildtype = 36.2±6.3 pA (n = 15); Y284C/+ = 18.0±1.6 pA (n = 12); Y284C/Y284C = 17.7±1.7 pA (n = 9); F = 4.72, *P = *0.017 by one-way ANOVA, post-hoc, wildtype *vs.* Y284C/+ and wildtype *vs.* Y284C/Y284C, *P*<0.05 by Tukey-Kramer test. These data indicate attenuation of the M-current in *Kcnq2* mutant mice.

## Discussion

### Advantages of the kick-in system

The kick-in system provides a solution for the problems associated with conventional knock-in technology. A comparison of both, the kick-in system and conventional knock-in technology, is shown in Figure 1. Conventional knock-in technology is a highly time- and cost-consuming undertaking that is primarily limited by resources, making the generation of multiple lines difficult. Any additional genetic alterations, even if situated near a previously examined change, require full-scale repetition of all previously invested effort, which complicates simultaneous analysis of many potentially interesting genetic variations. Side-by-side comparison of multiple knock-in mouse lines is therefore rare.

Contrary to the conventional knock-in technology, our kick-in system has been built on modified Cre/*lox* technology [21], [22]. The recombination efficiency of our system is ∼100% [11], whereas homologous recombination in the conventional strategy is as low as 1∶10^5^ [23]. The kick-in system first establishes ES cells with recombination sites enveloping the target genomic region. These acceptor ES cells then serve as a platform for the development of animals with genetic alterations within the target region. Maintenance of the acceptor ES cells requires no more than standard tissue culture procedures. Target region knock-in mice from the acceptor ES cells were obtained within 8 weeks. Development of genetically altered animals can therefore proceed at previously impossible rates, making the side-by-side comparison of multiple animal lines feasible. This constitutes a significant step forward for basic research, which, up to this point, focused mainly on examining a single, “most important” variant of a protein, one at a time.

The kick-in, of course, does not stand alone when it comes to improvements in knock-in technology. There are various other knock-in modifications that provide significant advances over the traditional knock-in approach, such as the CRISPR/Cas system [24] and TALEN [25]. The CRISPR/Cas approach, for example, allows generation of knock-in mice within 4 weeks [26], but it requires a Protospacer Adjacent Motif, which limits the boundaries of the knock-in, while the kick-in accommodates a large target region.

### Validity of the Kick-in System

Our mutant mouse lines demonstrate the validity of the kick-in system. We found no evidence of morphological or behavioral changes. Neuronal excitability, on the other hand, was elevated as revealed by EEG analysis and PTZ challenge. These findings compare to those gathered in mice produced with conventional *Kcnq2* knock-in technology. Singh *et al.* generated A306T mutant knock-in mice and examined them very closely. In their study, five A306T homozygous mice showed at least one forelimb and hindlimb tonic extension between P20 and P40, and all the mice died either immediately after birth or between P23 and P32 [9]. Although we did not observe spontaneous seizures in our A306T homozygotes, all of our mice similarly died at a young age. Interestingly, our Y284C homozygotes showed spontaneous forelimb and hindlimb clonus in addition to myoclonic seizures. Whether or not myoclonus can be a part of the phenotype in BFNE patients harboring the A306T or Y284C variation is uncertain, but patients with the A207T mutation reportedly do present with myoclonic jerks [27]. None of the Y284C homozygous mice died early, but 5 out of 17 (30%) homozygous mice perished during surgery for EEG analysis versus 3 out of 51 wildtype mice (6%) and 1 out of 50 heterozygous mice (2%), suggesting that Y284C homozygotic animals mice were more vulnerable during invasive brain surgery.

When challenged with the chemical convulsant PTZ, Y284C, and A306T animals showed elevated seizure susceptibility. These results are similar to the conventional knock-in A306T mice, in which the A306T heterozygotes had reduced electroconvulsive seizure thresholds, suggesting neuronal hyperexcitability [28]. Otto *et al.* proposed that increased seizure susceptibility and mortality after kindling relates to altered excitability in these animals [28]: PTZ and electroconvulsion both examine neuronal hyperexcitation, albeit through different pathways. Ties between neuronal hyperexcitability and *Kcnq2* deficiency are, of course, not new, as reported by Watanabe *et al.* who found hypersensitivity to PTZ [29].

In terms of neuronal hyperexcitability, A306T may produce stronger effects than Y284C, since A306T homozygotes died earlier than Y284C homozygotes. Direct comparative data between the human Y284C and A306T phenotype are not available [8], however, experiments in *Xenopus* oocytes revealed an *I_M_* reduction by about 90% in homozygous A306T, while homozygous Y284C *I_M_* was reduced by about 50%. Pseudo-heterozygous setups produce *I_M_* reductions approximating 40% in both cases [30]. It is possible that the pronounced *I_M_* effect is, at least in part, responsible for the premature death of the A306T homozygous animals. Singh *et al.* furthermore showed that CA1 neuronal *I_M_* amplitudes from A306T heterozygous mice were no different from those measured in WT mice, but were significantly decreased in A306T homozygous mice at every potential tested [9]. The researchers concluded that the decrease in induced seizure threshold without changes in current amplitude in the heterozygous knock-in mice might be the result of faster deactivation kinetics seen in heterozygous mice. We also conducted direct electrophysiological analysis on Y284C brain slices, which show a significant reduction in *I_M_* homozygous animals, in particular during strong depolarization at 4 and 10 weeks of age. Heterozygous brains also showed reductions in *I_M_*, which may relate to these animals’ lowered seizure threshold.

Since all experimental results confirm our approach’s functionality, we conclude that the kick-in is a suitable technique to quickly generate a wide variety of genetic alterations. We see the kick-in as an enabler for phenotyping efforts of novel pathogenic and non-pathogenic alterations in an era where new genetic variation data become rapidly available. We furthermore present a new BFNE model, which will help advance our understanding of pediatric epilepsy by providing a platform for various functional analyses.

## Supporting Information

Figure S1 **c-Fos based excitability measurements in the Kcnq2 mutants.** c-Fos expression is a measure of prior cellular activity. Wildtype somatosensory cortex and dentate gyrus were indistinguishable from that of newborn Y284C homozygotes. By comparison, close examination of the dorsal hippocampus, a region commonly implicated in seizure disorders, revealed a clear and significant increase in c-Fos expression, which is indicative of cellular hyperactivity (P<0.05, Tukey-Kramer test).
(TIF)
