# Correction: The Kick-In System: A Novel Rapid Knock-In Strategy

**DOI:** 10.1371/journal.pone.0106125

**Published:** 2014-08-13

**Authors:** The PLOS ONE Staff

Table 1 appears incorrectly in the HTML version of the article. Please see the correct Table 1 here.

**Table 1:** **Sequence information for lox sites.**

Name	Sequence
*lox*P	ATAACTTCGTATA GCATACAT TATACGAAGTTAT
*lox*71	TACCGTTCGTATA GCATACAT TATACGAAGTTAT
*lox*KMR3	ATAACTTCGTATA GCATACAT TATACCTTGTTAT
*lox*71/KMR3	TACCGTTCGTATA GCATACAT TATACCTTGTTAT
*lox*2272	ATAACTTCGTATA GGATACTT TATACGAAGTTAT
