# Supplementary figures and images for: The Kick-In System: A Novel Rapid Knock-In Strategy
Source: PLoS One. 2014 Feb 19;9(2):e88549. doi: 10.1371/journal.pone.0088549 (PMC3929540; doi:10.1371/journal.pone.0088549)

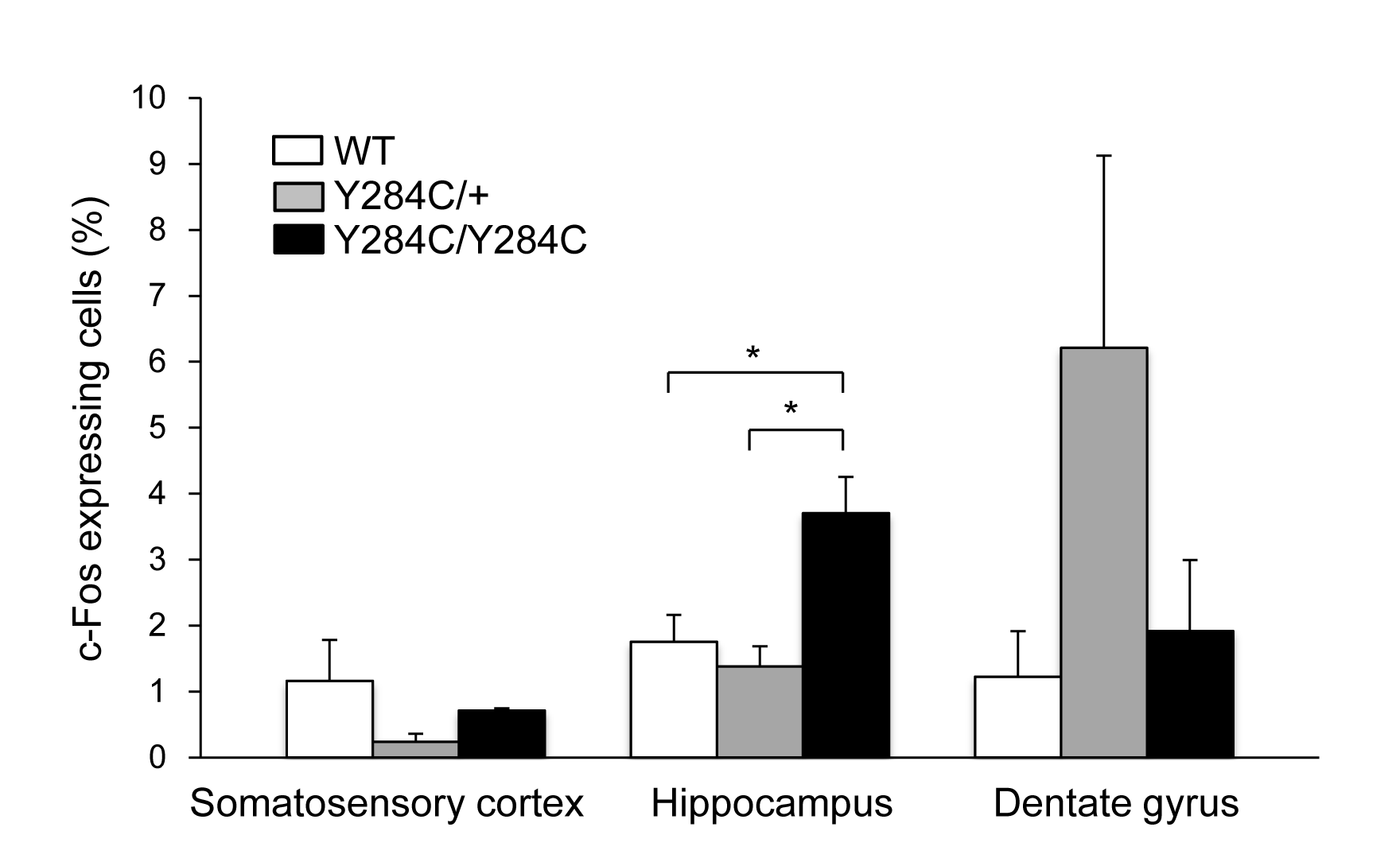

Supplement: Figure S1 — c-Fos based excitability measurements in the Kcnq2 mutants. c-Fos expression is a measure of prior cellular activity. Wildtype somatosensory cortex and dentate gyrus were indistinguishable from that of newborn Y284C homozygotes. By comparison, close examination of the dorsal hippocampus, a region commonly implicated in seizure disorders, revealed a clear and significant increase in c-Fos expression, which is indicative of cellular hyperactivity (P<0.05, Tukey-Kramer test). (TIF) [file pone.0088549.s001.tif]
